# Electric Field-Induced Nano-Assembly Formation: First Evidence of Silicon Superclusters with a Giant Permanent Dipole Moment

**DOI:** 10.3390/nano13152169

**Published:** 2023-07-26

**Authors:** Fatme Jardali, Jacqueline Tran, Frédéric Liège, Ileana Florea, Mohamed E. Leulmi, Holger Vach

**Affiliations:** 1 Laboratoire de Physique des Interfaces et des Couches Minces, CNRS, École Polytechnique, IP Paris, 91128 Palaiseau, France; fjardali0@gmail.com (F.J.); jacqueline.tran@polytechnique.edu (J.T.); frederic.liege@universite-paris-saclay.fr (F.L.); if@crhea.cnrs.fr (I.F.); 2 LMF, École Normale Supérieure, Paris-Saclay, 91190 Gif-sur-Yvette, France; 3 CRHEA, CNRS, Université Côte d’Azur, 06903 Sophia-Antipolis, France; 4 Center for SiNC Applications, 75000 Paris, France; mohamed.leulmi@siliconnanoclusters.com; 5 Centre for Research in Molecular Modeling, Concordia University, Montreal, QC H4B 1R6, Canada

**Keywords:** hydrogenated silicon nanoclusters, permanent electric dipole moment, self-assembled nanostructures, surface deposition, superclusters

## Abstract

The outstanding properties of silicon nanoparticles have been extensively investigated during the last few decades. Experimental evidence and applications of their theoretically predicted permanent electric dipole moment, however, have only been reported for silicon nanoclusters (SiNCs) for a size of about one to two nanometers. Here, we have explored the question of whether suitable plasma conditions could lead to much larger silicon clusters with significantly stronger permanent electric dipole moments. A pulsed plasma approach was used for SiNC production and surface deposition. The absorption spectra of the deposited SiNCs were recorded using enhanced darkfield hyperspectral microscopy and compared to time-dependent DFT calculations. Atomic force microscopy and transmission electron microscopy observations completed our study, showing that one-to-two-nanometer SiNCs can, indeed, be used to assemble much larger ”superclusters” with a size of tens of nanometers. These superclusters possess extremely high permanent electric dipole moments that can be exploited to orient and guide these clusters with external electric fields, opening the path to the controlled architecture of silicon nanostructures.

## 1. Introduction

The existence of hydrogenated silicon nanoclusters (SiNCs) with a permanent electric dipole moment and size of about one nanometer was theoretically predicted as early as 2005 [1]. Their outstanding stability has been traced back to extensive electron delocalization caused by the significant over-coordination of one or several of their constituting silicon atoms [2,3,4]. Ab initio simulations of the growth dynamics in a plasma reactor demonstrated the self-organized assembly of such SiNCs [5]. Possible applications were predicted in the field of photovoltaics and terahertz communication [6,7]. Recently, their permanent electric dipole moment was directly measured in a plasma reactor to be of the order of 2.2 Debye, in excellent agreement to prior theoretical predictions. Clusters with this relatively large dipole moment have consequently been used to reduce the work function of LaB${}_{6}$ cathodes employed, for example, in satellite thrusters. The simple presence of these SiNCs on a LaB${}_{6}$ cathode actually increased the thermionic electron density emission by a factor of up to 30. To this end, it was necessary, however, to deposit the polar SiNCs in a well-oriented manner, which was simply accomplished with the application of an electric field during their surface deposition on the cathode [8]. Interestingly, Schäfer et al. have very recently confirmed the existence of silicon clusters with a permanent electric dipole moment by means of electric molecular beam deflection experiments at cryogenic temperatures for clusters with 30 to 90 atoms [9], following the theoretical work of Jackson and Jellinek [10]. In this work, Schäfer et al. concluded that the dipole moment per atom is almost constant, indicating that the dipole moment should increase with cluster size. Our previous investigations, both theoretical and experimental, concerned quasi-monodisperse cluster size distributions with cluster sizes between 1 and 2 nm. Therefore, the work by Schaefer et al. immediately triggered our interest in the permanent electric dipole moments of much larger silicon clusters.

A silicon nanoparticle with a perfect crystalline structure (i.e., with all atoms being four-times coordinated) cannot have a permanent electric dipole moment. Therefore, we must restrict our search for large polar clusters to those that are not perfectly crystalline. However, it is not trivial to predict whether a cluster of a certain size will be crystalline or amorphous. There is a general consensus that growing silicon clusters undergo an auto-organized phase transition from that which is ill-defined to that of a crystalline morphology at a certain size, but it has not been possible to clarify at which size this transition takes place. On the one hand, it is well known that amorphous dusty clusters might be as large as hundreds of micrometers, while on the other hand, clusters as small as 2 nm have been observed to be crystalline. In this latter work, Kortshagen et al. chose plasma conditions in such a way that nanocrystal agglomeration was essentially suppressed, and the crystallization of even the smallest clusters was attributed to unconventional heating through electron–ion recombination processes [11].

To make sure that our nanoclusters do not have a crystalline structure, we thus adopted a scheme that is exactly contrary to the one of Kortshagen et al. Specifically, we created the same plasma environment as the one that already permitted us to produce one-to-two-nanometer amorphous-like SiNCs with a relatively strong permanent dipole moment [8]. Furthermore, we increased the on-time of our pulsed plasma from about 2 s to 8 s, which was sufficient time for the tiny SiNCs to agglomerate before the end of the plasma pulse. We will show that the resulting self-organized “superclusters” are, indeed, non-crystalline, and possess an unexpectedly large absorption spectrum as well as an extremely strong permanent electric dipole moment. This allows us to present the first experimental evidence of electric field-induced assembly formation with silicon superclusters.

## 2. Materials and Methods

### 2.1. Cluster Preparation

Since the experimental details have been published elsewhere [8], we will only concentrate on some major features here. Inside a 0.02 m${}^{3}$ vacuum chamber, an RF (13.56 MHz) CCP discharge was generated (Cesar 133 RF power supply) at low pressure (10 to 15 Pascals) between a grounded and a powered electrode. The circular electrode surfaces had a diameter of 7 cm and were separated by 4 cm. The shower head shape of the powered electrode allowed the injection of a gas mixture of silane (2%) diluted in argon (98%) directly between the electrodes, to produce hydrogenated silicon nanoclusters in a pulsed discharge. The flow rates of both the SiH${}_{4}$/Ar gas mixture and pure argon were controlled by mass flow controllers (MFC-Bronkhorst F-201DV, Low-ΔP-Flow), which were operated at typical flow rates of 10 sccm and 40 sccm, respectively. To also control the polar SiNCs in a horizontal direction (i.e., parallel to the substrate) before their deposition, a second set of vertical electrodes of 25 mm diameter was installed on both sides of the standard horizontal reactor electrodes.

The particle charging in low-pressure cold plasmas, like the one used in this work, has been extensively studied (see, for example, Fridman et al. [12]). It has been demonstrated, for instance, that all silicon particles undergo charge fluctuations in the plasma due to ion and electron bombardment occurring on their surfaces. For the decharging of dust nanoparticles in the plasma afterglow, it was shown that some particles keep a residual charge that has been evaluated to be 2% of the charge they acquired in the plasma [13,14]. For 3 nm SiNCs, it was proven that they never have more than one elementary charge in the plasma [12]. This means that all SiNCs considered here are neutral in the afterglow. We underline that we have already given experimental proof for this conclusion regarding our specific nanoclusters [8]: After their creation, our SiNCs had to travel a certain distance before laser detection. Even if they had been charged with only one single elementary charge, they should have traveled this distance within 9 μs because of the applied electric DC field. In the experiment, however, the majority of SiNCs only arrived after about 150 ms at the detecting laser beam. This travel time roughly corresponds to the time imposed by the gas flow. We can, thus, be sure that our SiNCs are neutral in the afterglow. Consequently, the electric DC field applied in the afterglow only acts on their permanent electric dipole moment.

As concerns the role of the SiNC charge in the plasma for the creation of a dipole moment, we have used ab initio methods to evaluate the influence of one elementary charge on their growth mechanism. It turns out that our 1 nm SiNCs only suffer minor deformations, but acquire a dipole moment that is considerably larger (about 4.0 Debye) than the one of their neutral counterparts (about 2.3 Debye). Therefore, the temporary charging of the SiNCs in the plasma actually facilitates the agglomeration for supercluster formation. We expect this mechanism to be operational until a supercluster size of about 50 nm in diameter. Thereafter, the electrostatic Coulomb repulsion is expected to overcome the kinetic energy of the colliding SiNCs [15].

We have previously demonstrated that we can totally neglect collisions between individual SiNCs in the time frame between the end of the plasma pulse and the moment of their surface deposition [8]. However, during the duration of the plasma on-time, the SiNCs are trapped in the plasma bulk and their probability of collision increases with the duration of the discharge. In our previous work, we have shown that a plasma on-time of 6 s or longer will strongly favor SiNC agglomeration. Therefore, we have chosen a discharge duration of 8 s for the present study. At 50 ms after the end of the discharge pulse, a DC bias voltage of 120 V or 50 V was applied either to the horizontal electrodes or to the vertical ones, respectively, to orient and guide the SiNCs in space before their deposition.

### 2.2. Enhanced Darkfield Hyperspectral Microscopy

CytoViva’s patented enhanced darkfield illumination performs two functions very differently from standard darkfield microscopes: First, it couples the source illumination directly to the condenser system, minimizing light loss. Second, it collimates the source illumination and focuses it precisely onto the condenser annulus. This maximizes the number of oblique angle photons focused on a very shallow focal plane. As a result, a significant increase in the signal-to-noise ratio detection capability of the darkfield is observed (up to 10-fold). In addition, a diffraction grating spectrophotometer is installed onto a microscope camera mount. It captures the unique reflectance spectra of objects from the microscope field of view in the wavelength region of about 400 nm to 1700 nm. The complete spectra for each pixel of the CCD detector are captured. Spectral data are reported in high resolution (down to 2.0 nm). The development of enhanced darkfield hyperspectral microscopy provides the ability to identify nanoscale materials in complex environments in a semi-quantitative manner. Hyperspectral images captured with the CytoViva system look similar to optical images. However, each pixel of a hyperspectral image contains the spectral response for the spatial area of that pixel. Using integrated hyperspectral image analysis software, the unique spectral responses of nanomaterials can be identified and easily mapped throughout the sample [16].

### 2.3. Atomic Force Microscopy (AFM)

For the acquisition of the AFM images, a commercial Bruker Dimension Icon in the PeakForce Tapping (PFT) mode was used. PFT is a Bruker-exclusive mode optimized to simultaneously achieve high-resolution images and quantitative mechanical measurements based on the deflection of the cantilever. During operation, the cantilever is brought in and out of contact with the surface in a non-resonant cycle, where the PFT algorithms directly control the instantaneous force interaction. Because the entire cycle is monitored and recorded, FT provides force spectroscopy information at each pixel. In the feedback mode, the instantaneous peak force is limited, potentially down to the piconewton level [17].

### 2.4. TEM, EDX, and Focused Ion Beam Analysis

Transmission electron microscopy (TEM), high-angle annular darkfield (HAADF), and energy-dispersive X-ray (EDX) spectroscopy analyses were performed on a Titan Themis transmission electron microscope operating at 200 kV equipped with a Cs aberration probe corrector and a Super X detector that allows chemical analyses of light and heavy elements with a spatial resolution within the nm range. In this case, a specific specimen focused ion beam (FIB) lamella preparation was required. Thus, cross-section lamellae were prepared using a standard lift-out procedure within a FIB dual beam microscope (FIB, FEI-Scios Dual Beam). For the chemical analyses, we chose, as main elements of interest, silicon (Si) with the K${}_{\alpha }$ = 1.74 KeV ionization edge and oxygen (O) with the K${}_{\alpha }$ = 0.523 KeV ionization edge. Carbon (C) and platinum (Pt) protective layers were deposited on top of the sample prior to the FIB milling process to prevent possible gallium (Ga) ion implantation during the milling process.

### 2.5. First Principles Calculations

In our previous works, we have demonstrated the reliability of the B3LYP functional combined with Grimme’s DFT-D2 dispersion correction scheme for our SiNCs in comparison to MP2 calculations [3,6] and experimental measurements [8]. Therefore, both structural optimizations and time-dependent density functional theory (TD-DFT) calculations were accomplished at a B3LYP + DFT-D2 level of theory using Gaussian G16 [18]. After all structural optimizations, frequency analyses were performed to assure that the optimized geometries really corresponded to minima on the corresponding potential energy surfaces. For the TD-DFT calculations, we always calculated 600 excited states leading to the shown absorption spectra. Since all computational details have already been published elsewhere [19], we only focus on some specifications here: To treat the relatively large cluster aggregates on the same level of theory as the individual clusters, we limited ourselves to the 6–31G(d,p) basis set, including dispersive interactions, as described by Grimme’s DFT-D2 method [20]. We underline that this approach led to a perfect agreement between the dipole moment for one SiNC, calculated here to be 2.28 Debye, and one previously measured to be 2.25 ± 0.25 Debye [8]. Nevertheless, we tested a possible influence of diffuse functions and a more recent Grimme correction scheme, but no major changes could be observed. For an individual SiNC, for instance, the inclusion of diffuse functions for both Si and H atoms only slightly decreases the dipole moment by 0.14 Debye. Replacing Grimme’s DFT-D2 method by his DFT-D3 method essentially gives the same results for the dipole moments within 1%, and reduces the total energy by less than 0.001%.

UV–Visible spectra are plotted as molar absorption coefficient (ϵ) vs. wavelength (λ). We have assumed that all absorption peaks possess a Gaussian band shape. The overall spectrum, ϵ(ν), as a function of the excitation energy, ν, then comes from the sum of all the individual bands, $\epsilon (\nu_{i})$:

(1) $$\epsilon \left(\nu \right)=\sum_{i=1}^{n}\left(B\times \frac{f_{i}}{\sigma }\times exp\left[-{\left(\frac{\nu -\nu_{i}}{\sigma }\right)}^{2}\right]\right)$$

where *i* runs from the first to the *n*th electronic excitation. In the present work, we have calculated the first 600 transitions for each absorption curve and assumed a value of 0.4 eV for the standard deviation σ. The constant *B* has a value of $1.3062974\times 10^{8}$ × L × eV × mol${}^{-1}$ × cm${}^{-1}$. The (dimensionless) oscillator strength is $f_{i}$ corresponding to the *i*th electronic excitation of interest with an excitation energy $\nu_{i}$.

## 3. Results

In our search for larger SiNCs with a permanent dipole moment, we started out with essentially the same experimental conditions as in our last work, but we increased the plasma on-time from 2 s to 8 s, which significantly favors the agglomeration of small one-to-two-nanometer SiNCs [8]. This agglomeration takes place under the influence of the ambipolar electric field naturally present in the plasma discharge [12]. This electric field assures that the orientation of the small polar SiNCs is the same as that of the growing supercluster during their agglomeration.

### 3.1. Bare-Eye Observation

**Figure 1:**
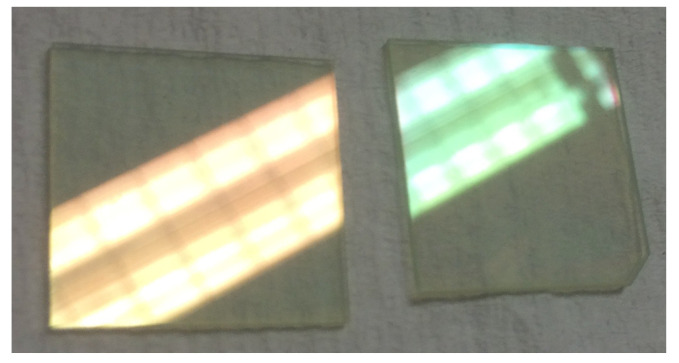
Photo taken after the deposition of two samples obtained with two orthogonal bias voltages (**left** side, horizontal E-field; **right** side, vertical E-field) with otherwise identical plasma conditions. Looking at the reflection of the room illumination, we can clearly distinguish two different colors.

For our first deposition, we used simple Corning glass substrates with sizes of approximately 50 mm × 50 mm. We deposited our plasma-born SiNCs during two succeeding runs with two orthogonal electric field directions. We achieved this by using our abovementioned two sets of electrodes, under otherwise identical deposition conditions. We used different times of deposition to control the layer thickness. Using ellipsometry, we determined that the deposited layer thicknesses of the samples shown in Figure 1 were about 50 nm, which excludes optical interference effects, in order to explain the difference in colors.

Instead, we propose that the deposition of SiNCs with two different, orthogonal dipole directions leads to thin films with different optical properties. To explore this hypothesis, we then employed a state-of-the-art procedure to measure the absorption spectra resulting from horizontally and vertically oriented SiNCs.

### 3.2. Hyperspectral Darkfield Absorption Measurements

For these measurements, we deposited our SiNCs onto 100 µm thick microscope glass slides (50 mm × 15 mm). To perform the planned enhanced darkfield hyperspectral microscopy measurements, we had to send our samples to Auburn, AL, USA. To protect our samples against possible oxidation during transport, we added a transparent 15 nm thick Teflon-like layer to cover them in situ using octafluorocyclobutane (C${}_{4}$F${}_{8}$) plasma directly after the SiNC deposition, before venting the reactor to the atmosphere. On site, our samples were then prepared by adding a drop of immersion oil onto a slide and placing the glass with the SiNCs directly on top. Hyperspectral images were acquired with transmitted enhanced darkfield illumination at 40× magnification. Data were recorded in the VNIR (400–1000 nm) range.

**Figure 2:**
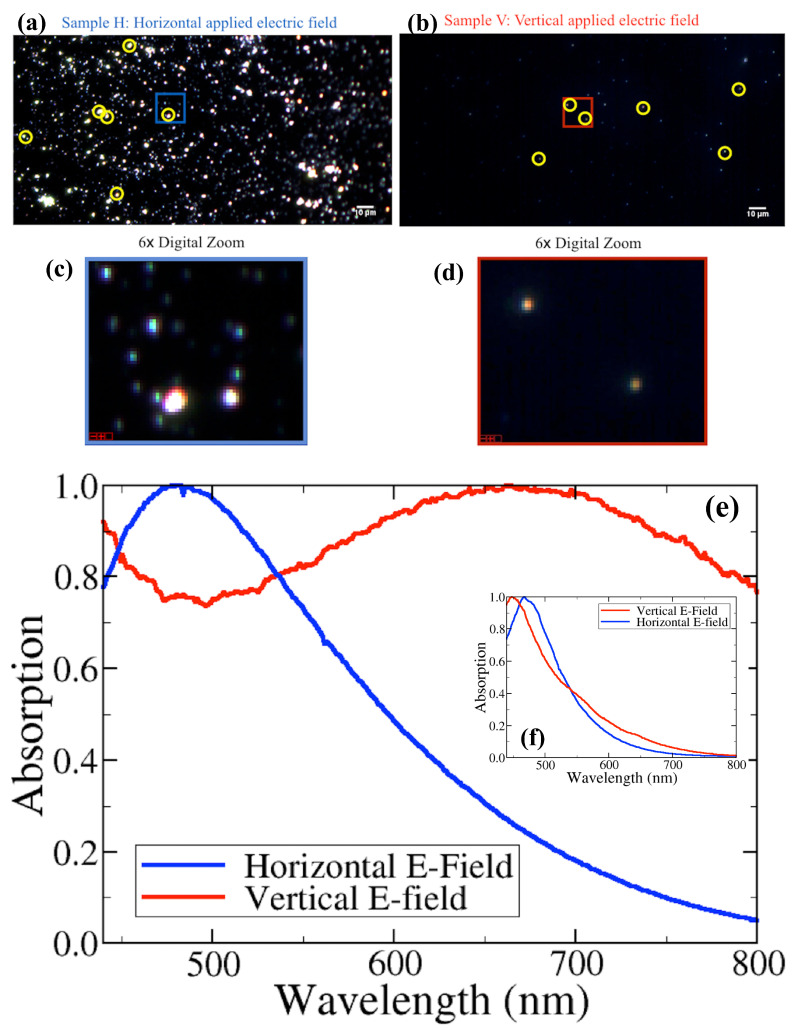
Darkfield hyperspectral imaging of dipole-oriented SiNCs deposited onto a glass substrate exhibiting unique spectral profiles. **Top panels** (**a**,**b**): hyperspectral images (taken at 40× magnification) of SiNCs deposited with a horizontal electric field (**a**) and a vertical electric field (**b**) applied during their deposition; six representative “regions of interest” (ROIs) are drawn around the chosen SiNCs (yellow circles). **Middle panels** (**c**,**d**): the selected SiNCs are shown at a higher magnification. **Bottom panel** (**e**,**f**): optical spectra measured from the ROIs defined above and averaged for mean comparison for both samples. Inset (**f**): normalized absorption spectra measured for the smallest observed SiNCs. **Main panel** (**e**): normalized absorption spectra measured for the largest SiNCs.

As can be seen in the hyperspectral images in the two top panels of Figure 2, there is a remarkable difference in the observed density of SiNCs for the two electric field orientations. This difference, however, has already been predicted based on the inhomogeneity of the applied electric fields in our setup. In fact, we have previously shown that the vertical electric field between our two horizontal electrodes does not only provide a torque that orients the molecular dipole moments, but that it also exerts a force on the polar clusters to displace them because of its inhomogeneity. It actually turns out that this field “steers” a considerable fraction of our SiNCs toward a “magic ring” that has a diameter of about 6 cm [8]. Our samples, however, were placed in the very center of the cathode during depositions, explaining the relatively low SiNC density for the vertical E-field polarization. This striking difference in SiNC density for the two orthogonal E-fields already manifests convincing evidence that SiNCs produced under the present plasma conditions possess a significant permanent electric dipole moment—plasma conditions that are prone to lead to significantly larger agglomerated SiNCs than those in previous works [8,9].

Despite the relatively small number of SiNCs appearing for vertical polarization, there were enough of such clusters to pursue our optical absorption measurements. We have actually been able to perform two distinctively different nanocluster analyses: one for relatively small SiNCs (see inset of Figure 2) and one for relatively large SiNCs (see main bottom panel of Figure 2). Note, that we will address the question of absolute cluster size in Section 5. While the absorption spectra recorded for the small SiNCs are reasonably comparable for both E-field polarizations, there is an unexpectedly important difference for the large ones: the vertical E-field (V) leads to a much broader absorption spectrum, and the maximum absorption wavelength is shifted across the entire visible wavelength region by more than 200 nm relative to the one corresponding to the horizontal field (H). To assure that the H sample does not exhibit peaks as broad as those of the V sample, a Peak Location Classifier algorithm [21] was run to find any particles with peaks of 700 ± 100 nm, but none were found. It appears as though H may have activity in the UV region, whereas V is fairly active throughout the entire visible region until the near-infrared region.

**Figure 3:**
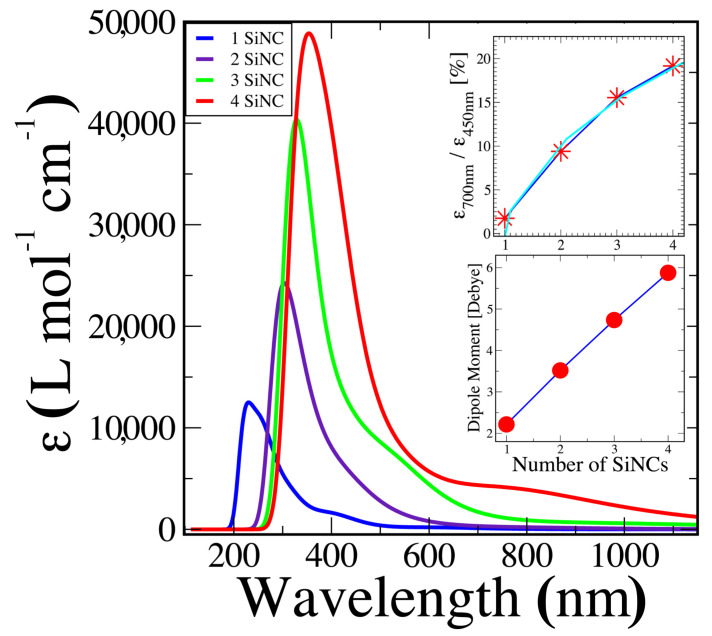
Theoretical molar absorption coefficient ϵ as a function of wavelength for clusters self-assembled from one to four of the most stable, presently known, individual 1 nm SiNCs (see text). **Upper inset**: absorption intensity ratio at the wavelengths roughly corresponding to the experimentally observed absorption maxima as a function of cluster size; the cyan curve corresponds to a logarithmic regression. **Lower inset**: calculated permanent electric dipole moments as a function of cluster size.

To address the question of how SiNCs deposited with a vertical E-field can have such a broad absorption profile, and specifically, how they can so efficiently absorb light in the red and even in the near-infrared spectral region, we realized a series of ab initio simulations. As we have seen in Figure 2, the absorption spectra not only show a crucial dependence toward the applied E-field direction, but also on the size of the deposited SiNCs. Therefore, we calculated the absorption spectra for SiNCs with four different sizes. To this end, we placed between two to four SiNCs in close proximity and optimized the geometries of the resulting ensembles by minimizing their energy. In all three cases, the lowest-energy structures showed one covalent bond between neighbor clusters. With time-dependent density functional theory (TD-DFT), we then calculated the first 600 transitions for each aggregate, and determined the corresponding molar absorption coefficient ϵ as a function of wavelength, as described in detail above. The results are shown in the main panel of Figure 3.

As expected, the overall absorption capacity increases with the number of aggregated SiNCs. It appears that the spectra are composed of two components: one that is dominant in the UV region and one that shifts toward longer wavelengths and increases its relative importance as the aggregate size becomes larger. It is obvious, however, that we need much larger aggregates to reproduce the experimentally observed intensity ratio between these two components, as displayed in Figure 2 for the vertical E-field. To obtain at least an order of magnitude estimate of how large such an aggregate should be, we have traced, in the upper inset of Figure 3, the intensity ratio as calculated at the wavelengths roughly corresponding to the two experimental absorption maxima. Unfortunately, we have too few points for a reliable extrapolation. As a highly conservative estimate, we first calculated a linear regression curve, which suggests that we need at least 12 SiNCs (i.e., about 228 silicon atoms) to come to the experimentally observed ratio of about 0.92. However, a logarithmic regression on the same four points (see the cyan line) shows a much better fit, and suggests a minimum aggregate size of about 2400 SiNCs (i.e., 46,000 silicon atoms). While the latter fit agrees much more with our four data points, we can only qualitatively conclude at this point that we need much larger aggregates than those accessible for our present ab initio calculations to explain the measured absorption curve for the vertical polarization. In Section 5, we will actually measure the aggregate size that is necessary for the observed spectra, and will compare it to the present estimates.

In the lower inset of Figure 3, we have displayed the permanent dipole moment calculated for the four investigated structures. As recently suggested [9], we also found a nearly linear increase in the dipole moment with aggregate size (i.e., number of silicon atoms). Different to reference [9], however, we are not here dealing with pure silicon clusters, but with hydrogenated silicon clusters; i.e., instead of having a dipole moment of about 0.02 Debye/Si atom, typical of pure silicon clusters, we report ∼0.08 Debye/Si atom. In the present calculations, we always had one covalent bond between all clusters. As we will see below, this dipole moment per silicon atom will become even more important for SiNCs that are held together only by non-covalent bonds.

### 3.3. Atomic Force Microscopy Observation

**Figure 4:**
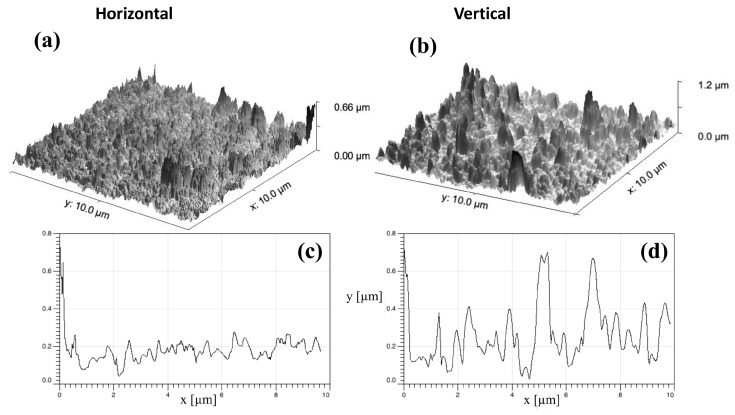
**Top panels** (**a**,**b**): three-dimensional representation of two AFM scans of two typical samples deposited with two orthogonal polarizations of the DC bias during deposition with otherwise identical plasma conditions. **Lower panels** (**c**,**d**): profile scans of above samples in two randomly chosen directions. Note that the overall roughness is about six times higher for the vertically than for the horizontally applied electric field deposition.

After having explored some of the optical properties that depend on the orientation of the permanent electric dipole moments of our deposited SiNCs, we investigated how this E-field programmable SiNC orientation can influence the mechanical properties of a substrate. In Figure 4, we show the AFM images resulting from two subsequent depositions of our SiNCs on intrinsic silicon substrates with two orthogonal E-fields applied during depositions with otherwise identical plasma conditions.

While the average peak height for the horizontal E-field polarization is obviously much lower than for the vertical one, some higher peaks do appear at the right edge of the sample. We tentatively attribute the appearance of these peaks to the breakdown of the homogeneity of the horizontal E-field. Nevertheless, even including these relatively high edge peaks, the sample deposited with V-polarization exhibits vertical structures that are, on the average, six times higher than the structures of the sample deposited with H-polarization.

As we have previously shown, the inhomogeneity becomes strongest at the edges of the electrodes [8]. For the horizontal polarization, this effect becomes further amplified by the fact that the electrodes are much smaller and further apart than for the vertical polarization. In addition, for the V-polarization, SiNCs that start their journey in the very center of the electrode will experience a perfectly homogeneous E-field until their surface deposition. For the H-polarization, however, the same SiNCs must cross the most inhomogeneous part of the H-field twice on their way to the substrate, potentially causing some misalignment among them. Therefore, the H-polarization cannot align the dipole moments as precisely as the vertical one can, in our present setup. As a result, the steering and orienting capacity are less efficient for the H-polarization, leading to smaller ensembles and no apparent horizontal structures lying on the substrate within the limited lateral resolution power of the AFM. As a consequence, these much smaller structures cannot efficiently absorb in the red region, as we can see in Figure 2.

### 3.4. TEM, STEM-HAADF, and EDX Observations

**Figure 5:**
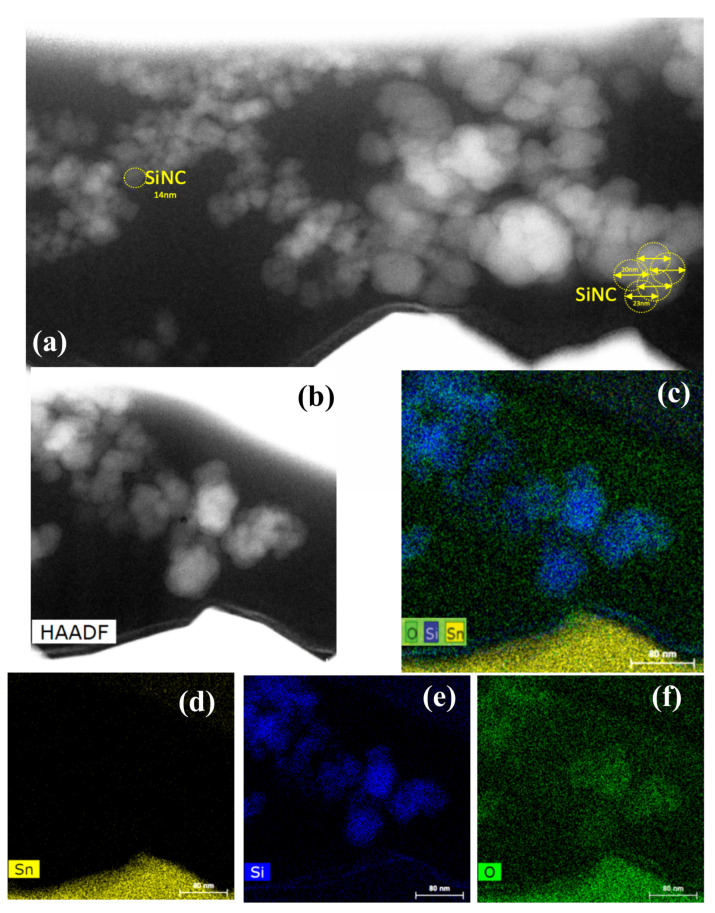
**Top panel** (**a**): STEM-HAADF image of SiNCs deposited onto an indium tin oxide (ITO) substrate together with an approximate diameter estimate. On top of the image, the carbon and platinum protective layers can be seen. **Middle panel** (**b**,**c**): STEM-HAADF images of the same silicon clusters and their corresponding EDX mapping with silicon (blue), oxygen (green), and tin (yellow). **Bottom panel** (**d**–**f**): the elemental EDX maps for the selected elements.

Finally, we address the question of how large the deposited clusters are, which shape they possess, and whether they are oxidized or not. To this end, we simultaneously deposited our SiNCs onto an ITO substrate and a TEM grid (“holey” carbon Cu 300 mesh). To visualize the SiNCs deposited onto the ITO substrate, we first tried to “scratch” off some material with a scalpel for the TEM analysis. However, we did not succeed in doing so because the deposited layer was too resistant. Therefore, we employed the *focused ion beam* (FIB) technique, as described above. In Figure 5, we can distinguish an impressive quantity of SiNCs ranging in size from about 4 nm to 20 nm, with an average size of about 14 nm. There seem to be some even larger clusters, but careful inspection suggests that they are the result of agglomeration of smaller ones. Note that a spherical cluster with a 14 nm diameter could result from the agglomeration of about 2700 SiNCs, each ∼1 nm in diameter. This estimate agrees surprisingly well with our logarithmic regression, shown in the upper inset of Figure 3, that suggests an aggregate size of 2400 SiNCs. A qualitative EDX analysis of the same sample suggests that the level of silicon cluster oxidation is reasonably marginal, and seems to be limited to the outer surface of the aggregates. Performing electron diffraction, we did not observe any diffraction spots or rings underlining the non-crystalline structure of our superclusters.

**Figure 6:**
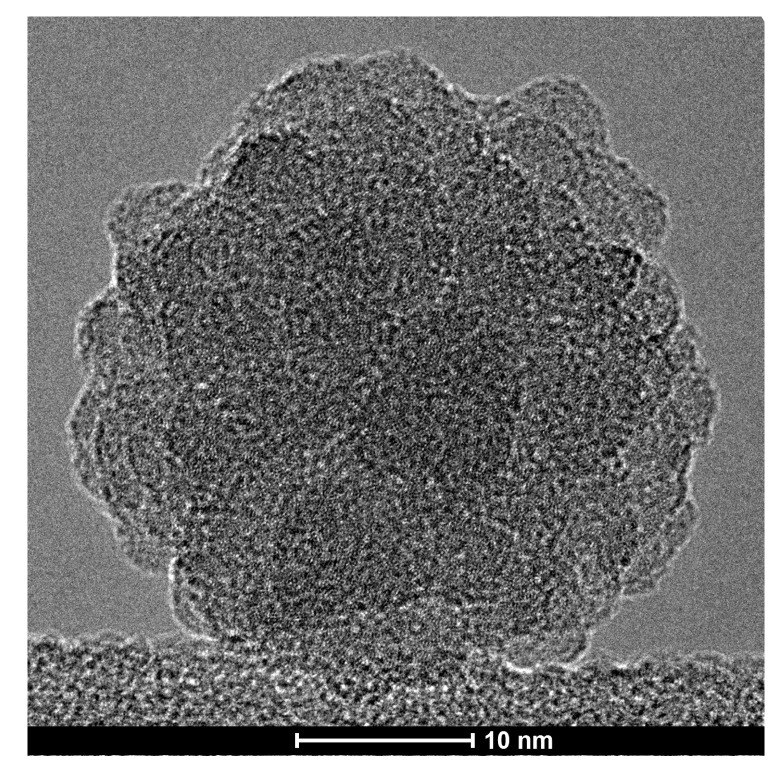
High-resolution TEM image of a typical nearly spherical silicon supercluster deposited onto a TEM grid.

In Figure 6, we present a high-resolution image of a 20 nm SiNC deposited onto a TEM grid resulting from the same deposition run. Its shape appears rather spherical, and it seems that it is, indeed, composed of many smaller SiNCs.

**Figure 7:**
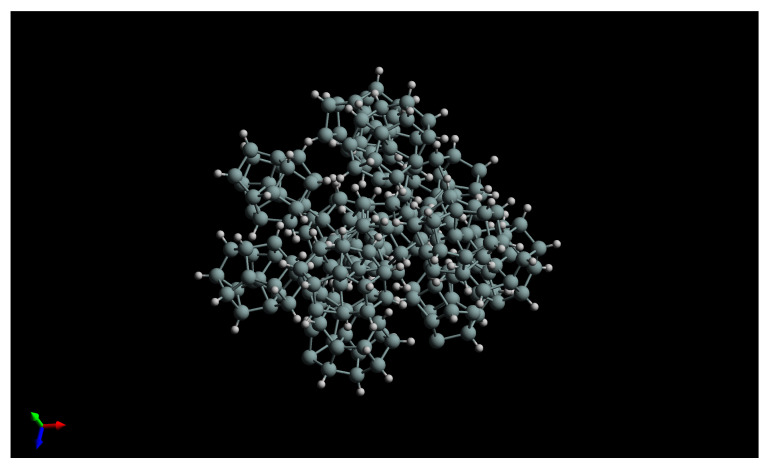
Ab initio simulation of a supercluster composed of 12 SiNCs with only two covalent bonds between all individual SiNCs, resulting in a theoretical dipole moment of 35 Debye.

Figure 7 shows the results of an ab initio simulation conducted in order to have a rough idea of what a cluster composed of several individual SiNCs looks like, and which kind of dipole moment we could expect. To this end, we placed 12 SiNCs in close proximity and let the electronic structure program optimize the ensemble to obtain a supercluster. The SiNCs we used in the present simulations were not fully passivated [22] which, fortunately, seems to describe our experimental SiNCs rather well. In the present case, two covalent bonds formed between three neighboring SiNCs during the structural optimization process, as we had already seen in our four-SiNC study described above. Nevertheless, there are still nine individual SiNCs that remain strongly bonded to the supercluster by only non-covalent bonds. Despite the two covalent bonds, the shown supercluster possesses a permanent electric dipole moment of 34.6 Debye. This roughly corresponds to 0.150 Debye per silicon atom, which is more than we found above for the four SiNCs, simply because covalent bonds were formed between all four SiNCs. Actually, this dipole moment per silicon atom turned out to be higher than for an individual SiNC (i.e., 0.118 Debye per silicon atom), attributable to induced dipole moments that have to be added to the initial permanent electric dipole moment. We can thus confirm the suggested quasi-linear dependence of the dipole moment value on the number of silicon atoms at least up to 228 atoms.

## 4. Discussion

Molecular entities with giant permanent electric dipole moments have an uneven distribution of electric charge, which gives them unique properties. For example, they tend to have high polarity, which makes them useful in solvents, as they can dissolve polar substances. They can also be used as building blocks for materials with specific properties, such as high dielectric constants, which are important in electronic devices. In addition, entities with large dipole moments can have important implications for biochemistry, as they can interact with biological molecules such as proteins and nucleic acids, affecting their structure and function. Other potential applications include material work function tailoring, nonlinear optics, ferroelectrics, and organic photovoltaics.

The search for molecules with giant permanent dipole moments is, consequently, a very active field of research. Recently, 5,6-diaminobenzene-1,2,3,4-tetracarbonitrile was unveiled because, with a dipole moment of 14 Debye, it has the highest known dipole moment of any neutral molecule [23]. A yet higher dipole moment was shown by a rather exotic species: a trilobite Rydberg dimer, a highly excited type of two-atom molecule $Cs_{2}$ with its atoms 100 nm apart; it was found to have a dipole moment of thousands of Debye, albeit under a high vacuum at just 40 µK [24].

In the present work, we used plasma conditions that are reasonably comparable to our previous work, in which we slowed down the reaction dynamics in a plasma reactor in such a way that polar SiNCs, with sizes of roughly one to two nanometers and permanent dipole moments of about 2.25 Debye, were formed [8]. In contrast to our previous work, however, we lengthened the duration of the plasma discharge here from 2 s to 8 s, which allows the rather-well-passivated 1–2 nm SiNCs to agglomerate. As a result, we are working here with aggregates that have sizes of roughly 20 nm.

During the supercluster growth by agglomeration, the ambipolar electric field plays a crucial role. In general, this field points from the center of the plasma to the electrodes or the walls. Specifically, the field points toward the upper electrode for the upper half of the plasma and toward the bottom electrode for the lower half. However, wherever the growing supercluster “picks up” another tiny polar SiNC, the ambipolar electric field naturally occurring in the discharge [12] assures that the two dipole moments of the two agglomerating entities possess the same orientation, causing the permanent electric dipole moment of the supercluster to increase with each newly attached SiNC. After the plasma is switched off, the ambipolar field disappears immediately and the strongly polar supercluster is oriented (and guided) by the then switched-on applied DC bias, either between the set of vertical or horizontal electrodes.

In this sense, we define a “supercluster” as a nanoscale entity that has been constructed by the agglomeration of one-to-two-nanometer polar silicon clusters with their permanent dipole moments directed in the same direction due to the ambipolar electric field of the plasma discharge during its creation. The precise plasma conditions determine to which degree the small SiNC building blocks are passivated with hydrogen atoms. Perfectly passivated SiNCs will lead to the highest possible permanent dipole moment for the resulting supercluster, while non-passivated silicon atoms might form covalent bonds between the small building blocks, increasing the overall stability of the ensemble. We underline, however, the importance of non-covalent forces between our SiNCs. For the above example of 12 SiNCs, the binding energy resulting for perfectly passivated SiNCs (i.e., without any inter-cluster covalent bonds) can readily be calculated to be approximately 6.78 eV. This means that (even for the ultimate case of absolutely no covalent bonds between the individual building blocks) the proposed superclusters are expected to remain stable even at very high temperatures because of the strong dipole–dipole interaction between all SiNCs due to their very close proximity.

Based on the measured absorption spectra shown in Figure 2, we can readily understand the colors observed in Figure 1. For the horizontal polarization, the absorption peak appears around 480 nm, which leads to a complementary orange/red color for the reflected light. For the vertical polarization, however, the main absorption peak is located around 700 nm, which leads to a reflection in the green [25].

There is a very important conclusion we can draw from the measured absorption spectra shown in Figure 2. Comparing the field dependence of absorption for the smallest and the largest SiNCs, it is obvious that the absorption spectra of large SiNCs depend much more strongly on the E-field polarization than those of smaller ones. Consequently, we can qualitatively conclude that larger SiNCs have stronger dipole moments than smaller ones. This, however, can only be true if our large clusters do not possess a crystalline structure. Straightforward experimental evidence for this conclusion is given by the absence of any diffraction spots or rings during our TEM analysis. The non-crystalline nature, even for our largest clusters, can become possible if they are assembled from non-crystalline 1–2 nm SiNCs, such as those of our previous study [8]. The cauliflower-like structure of a 20 nm supercluster, as shown in our TEM image in Figure 6, supports such a hypothesis.

For the absorption spectra shown in Figure 2, we observed the appearance of a second spectral component that increases its importance and progressively shifts toward the infrared region as the number of agglomerated SiNCs increases. As we point out elsewhere, we must remember that delocalized electrons are less tightly bonded to their chemical structures than localized ones. Therefore, it is well known that the absorption spectra of chemical compounds shift to longer wavelengths as their degree of aromaticity increases [6,25], indicating that the degree of electron delocalization might increase with the size of the supercluster. Such an increase, however, could, in principle, decrease the expected permanent dipole moment because the electron density becomes more evenly distributed throughout the cluster, resulting in a more balanced distribution of charge and a smaller overall dipole moment. In our present case, however, we believe that this effect is relatively negligible because the position of the maximum absorption, corresponding to this spectral component, appears at about 700 nm for both the four-SiNC ensemble (as calculated in Figure 3) and for a supercluster of about 2400 SiNCs (as measured for the vertical polarization in Figure 2).

From the TEM images in Figure 5, we can estimate that the average aggregate size is about 14 nm (i.e., there are roughly 51,000 silicon atoms or the equivalent of 2700 SiNCs 1 nm in size). This observation agrees remarkably well with our estimate of 2400 SiNCs based on the logarithmic regression shown in the upper inset of Figure 3. We point out that the samples analyzed in Figure 5 were exposed to ambient air after their deposition for 6 years prior to the shown measurements. Individual one-to-two-nanometer SiNCs deposited at the same time, also exposed to air, totally disappeared due to oxidation in the same time interval. This observation can clearly be understood by the fact that 1–2 nm clusters do not have any volume, and nearly all silicon atoms are located on the surface of the cluster (i.e., once these surface atoms are oxidized, there is no silicon cluster left). For the 20 nm superclusters, however, we have shown that the oxidation apparently only took place on the outer surfaces, leaving the majority of the inner 1–2 nm aggregated and aligned SiNCs intact. We can safely assume that this surface oxidation corresponds to the well-known formation of a native oxide layer with a typical thickness of about 2 nm. It is this oxide layer that protects the inner 1–2 nm SiNCs, which should largely conserve their individual properties if they are sufficiently well passivated.

## 5. Conclusions

To date, there have only been two experimental reports confirming the theoretically predicted permanent electric dipole moments of small silicon clusters [8,9]. While the technique of laser evaporation is very powerful [9], we tentatively believe that our pulsed plasma approach could be more efficient in creating clusters with large dipole moments; this is because the naturally present ambipolar electric field in the plasma aligns the dipole moments of the small SiNCs during agglomeration. Wherever the growing supercluster “picks up” another small SiNC in the plasma, the naturally occurring ambipolar electric field assures that the two dipole moments possess the same orientation. This causes the permanent electric dipole moment of the supercluster to increase with each newly attached SiNC. For the laser evaporation technique, however, there seems to be a risk that the randomly oriented electric dipole moments created between under- and over-coordinated silicon atoms might cancel each other out once a certain cluster size is exceeded because of the absence of any atomic alignment of the randomly oriented dipole moments.

With our method, it is the ambipolar electric field in the plasma discharge that aligns the dipole moments and enables the creation of superclusters with giant permanent electric dipole moments, as we have seen in Figure 2. Thereafter, it is the electric field that we only switch on after the supercluster creation (i.e., when the pulsed plasma discharge is switched off) that orients the supercluster on its way to the substrate. In the future, more homogeneous electric fields will help us to align the permanent dipole moments of the superclusters even more precisely before surface deposition to assure that they are arriving perpendicularly or at a well-defined angle to the substrate. With more sophisticated control of the inhomogeneities of the employed electric fields during deposition, one can realize the building of ingenious nanostructures in the future based on the here-demonstrated electric field-induced assembly formation. In this sense, the externally applied electric fields will assure the nano-architecture on a sub-micrometer scale, while the inherent dipole–dipole interaction between the superclusters assures the building block alignment on an atomic scale. The precise sizes of our supercluster building blocks are readily controlled by the duration of the plasma discharge, while the dimensions of our assembled nanostructures are controlled by the duration of the surface deposition.

At this point, however, the dipole moments of our superclusters remain to be measured, as we have only presented qualitative experimental evidence that the dipole moments of the superclusters should be significantly higher than the ones of individual SiNCs, which have previously been measured to be 2.25 Debye. This experimental challenge, however, should be motivated by our present computational prediction that a small supercluster of only 12 SiNCs should already have a dipole moment of 35 Debye. The experimental feasibility of such superclusters with PECVD plasma is straightforward, as can be seen in Figure 5 and Figure 6. Their dipole moments before surface deposition can either be measured by analyzing their trajectories in electric fields [8] or with time-of-flight techniques [9]. After their surface deposition, scanning tunneling microscopy (STM) can, for instance, be used to measure their dipole moments by analyzing the changes in the tunneling current as the tip is moved over the cluster. There is no theoretical reason why the addition of dipole moments should saturate above a certain value. However, if the dipole moments of individual SiNCs continue to add up, then the experimentally observed 14 nm clusters would be expected to have dipole moments greater than 6000 Debye, which would even eclipse the present world record of the exotic cesium dimer excited to a Rydberg state in a vacuum at 40 µK [24]. It all depends on which length scale the atomic-scale alignment precision of the electric dipole moments can be preserved, which would open an exciting path to more in-depth studies of our demonstrated proof of concept. Ultimately, the giant permanent electric dipole moments of our silicon superclusters can be exploited in many practical applications, for instance, as a new ferroelectric material, in nonlinear optics, or even for certain catalytic reactions.

We conclude with an extension inspired by our present work: It is well known that hydrogenated amorphous silicon (a-Si:H) grown in a plasma-enhanced chemical vapor deposition (PECVD) reactor can contain over-coordinated and under-coordinated silicon atoms, which contribute to the disorder and structural heterogeneity of the material. Similar to the small silicon clusters created by Schäfer et al. [9], we propose that dipole moments are created between these over- and under-coordinated silicon atoms. However, due to the random nature of amorphous silicon, these randomly oriented dipole moments cancel each other out over a sufficiently large piece of material. Therefore, we highlight the need to explore the possible creation of a well-oriented permanent electric dipole moment for an amorphous silicon film, grown under the influence of an homogeneous DC electric field. Once again, in this instance, the possible success depends on how well the individual dipole moments can be aligned with atomic precision.

## Data Availability

Additional data and information are available upon request to the authors.

## Abbreviations

The following abbreviations are used in this manuscript:

SiNC	Silicon Nanocluster
AFM	Atomic Force Microscopy
TEM	Transmission Electron Microscopy
STEM-HAADF	Scanning Transmission Electron Microscopy—High-Angle Annular Darkfield
EDX	Energy-Dispersive X-ray spectroscopy
FIB	Focused Ion Beam
ROI	Region Of Interest
TD-DFT	Time-Dependent Density Functional Theory
